# Dealing With Rejection: An Application of the Exit–Voice Framework to Genome-Edited Food

**DOI:** 10.3389/fbioe.2019.00057

**Published:** 2019-03-22

**Authors:** Bartosz Bartkowski, Chad M. Baum

**Affiliations:** ^1^Department of Economics, UFZ–Helmholtz Centre for Environmental Research, Leipzig, Germany; ^2^Institute for Food and Resource Economics and Bioeconomy Science Center, University of Bonn Bonn, Germany

**Keywords:** CRISPR, exit and voice, food innovation, food labeling, genome editing, governance, public deliberation, D11, D18, O38, Q18, Q58

## Abstract

Genome editing has been hailed as both a revolutionary technology and potential solution to many agriculture-related and sustainability problems. However, owing to the past challenges and controversy generated by widespread rejection of genetic engineering, especially once applied to agriculture and food production, such innovations have also prompted their fair share of concern. Generally speaking, much of the discussion centers on the inadequacy or uncertainty of current regulatory regimes, partly owing to the vastly different approaches in the European Union and United States. Insofar as this focus on regulatory regimes is stimulated by the desire to bridge the divide between proponents and critics of genome editing, it risks losing sight of an essential aim of regulatory action: effectively responding to and fostering trust in consumers and the public. In this article, we thus assign priority to understanding the contours of individual dissatisfaction and its related responses. Toward this end, we apply and extend Hirschman's exit–voice framework to bring together, synthesize, and give much-needed substance to the diverse expressions of dissatisfaction and discontent with novel genome-editing technologies. Through the resulting synthetic framework, we then identify and evaluate which governance approaches can prevent actions seen to be problematic and, moreover, open up the space for a more active public. In this context, we devote specific attention to (i) use of labeling as a means to enable “exit” of consumers from markets and (ii) public deliberation as a possible expression of “voice.” Indeed, both options are proposed and utilized in the context of genome editing, e.g., as a way for skeptical consumers to express their viewpoints, seek change in prevailing food systems, and navigate the conflicts and tensions from applying unique sets of values to assess the balance of risks and benefits. So far missing, though, is an evaluation of how well such efforts offer effective means for public expression, which is why we also link this framework to the wider issue of consumer sovereignty. Having done so, we conclude with a brief commentary on the potential and limitations of both options in the existing institutional framework of the EU.

## Introduction

Genome editing has been hailed as a revolutionary technology and the potential solution to many agriculture-related and sustainability problems (Baltes et al., 2017; Zilberman et al., 2018a). The new possibilities offered by genome editing, particularly via novel methods like CRISPR-based systems, however, also entail that existing governance solutions for genetically modified (GM) food are rendered (at least partly) obsolete. It thus becomes unclear how applications of genome editing in the food sector *should* be governed and regulated, or whether any special regulation is in fact necessary at all. Multiple opinions on this subject have already been voiced (e.g., Araki and Ishii, 2015; Huang et al., 2016; Kuzma, 2016; Malyska et al., 2016; Pollock, 2016), and even more in the wake of the recent judgment by the European Court of Justice that “all organisms obtained by mutagenesis,” even those resulting from genome editing, are identical in terms of the associated risks to health and environment (ECJ, 2018). Regardless of underlying differences in the process involved, and in contrast to the approach set forth by the relevant authorities in the United States (Waltz, 2016; USDA, 2018), use of any mutagenic^1^ technique to alter genetic material “in a way that does not occur naturally by mating and/or natural recombination” is now likely to require the same level of regulatory scrutiny across the European Union (ECJ, 2018).

Herein we find vivid expression of perhaps the central divide in the literature on the regulation of genome-editing products: between so-called product- and process-based approaches. In specific, the former often assigns priority to scientific risk assessments, thereby resulting in an emphasis on the “substantial equivalence” of products derived from genome editing with those engendered by “natural” processes. According to this approach, the prevailing criterion for deeming a product to be safe, whether developed by conventional breeding, genetic engineering, or genome editing, is whether it has a substantially different effect on a range of outcomes, e.g., human health and environmental impact, as compared with products available on the market (OECD, 1993; USDA, 2018). However, if there is no evidence for any such differences, then the products should be viewed identically from a regulatory point of view, irrespective of the breeding approach applied. Recently, in fact, the USDA (2018) (implicitly) upheld the determination of “substantial equivalence,” pointing to the fact that no foreign DNA had been inserted as a reason that CRISPR-based systems did not require “special” regulatory oversight.

On the other hand, process-based approaches assign more specific attention to whether there are fundamental differences with explicit respect to the underlying processes themselves. In the case of genome-editing techniques, with CRISPR-based systems currently occupying the cutting-edge here (Brinegar et al., 2017), it is both the greater precision to make changes at a specified location in the target DNA—thus the use of “editing”—and the combinatorial capacity to simultaneously enable many such changes (multiplexing; Barakate and Stephens, 2016) that render them distinct from “conventional” genetic engineering. At the same time, there is quite recent evidence that the CRISPR-based systems might also result in unwanted deletions and complex rearrangements of DNA (Kosicki et al., 2018) and that cells edited using such systems could be more susceptible to cancer (Haapaniemi et al., 2018). Given the growing (scientific) evidence of a connection between such problems and the underlying processes, this provides one argument supporting a more process-based approach (i.e., given that the nature of the effects extends beyond those for changes to a single product or product characteristic). Moreover, it has been argued that such an approach could provide greater scope to better consider issues such as the potential for consumer acceptance, perceived “naturalness” of biotechnology (Hartley et al., 2016; van Hove and Gillund, 2017; Pirscher et al., 2018), or the rate at which modifications occur (ECJ, 2018) when assessing the possible risks. Taking a step beyond the assessment of risks in (controlled) real-world settings, such an approach would highlight the wider relationship between technologies and the social, scientific, and technological contexts in which they would be applied (see Sjöberg, 2002). Currently, such a broad understanding of what constitutes “risk” is rarely taken into consideration within most prevailing regulatory approaches for GM food.

Given the nature of recent developments in the domain of genome editing, and the resulting rise in regulatory uncertainty, a novel analytical framework is necessary to synthesize and reconcile these disparate perspectives. In this regard, this article seeks to venture beyond the extant debate about, e.g., if the regulatory approach in the EU is justified and whether genome-editing should not also be entitled to a “mutagenesis exemption” (Purnhagen et al., 2018). Instead, we highlight that, for better or worse, whenever a country has decided against giving free rein to the products of genome editing and/or genetic engineering, this is prompted by the expressed discomfort and anxiety of large swaths of the general public (e.g., Gaskell et al., 2010; Hess et al., 2016; Cui and Shoemaker, 2018). What is required, as a result, is a deeper engagement with the public, which is itself predicated upon a greater understanding of the contours of individual dissatisfaction and its related responses. To facilitate this, we first bring together, synthesize, and give much-needed substance to the ways in which people express discontent with new genome-editing technologies. And second, through the resulting synthetic framework, we can then identify and evaluate which governance approaches can prevent actions seen to be problematic and, moreover, open up the space for a more active public to express criticism or support. In other words, looking past trade-offs between the potential benefits of genome editing and widespread opposition to genetic engineering, we wish to explore whether facilitating a more active role for the general public in regulatory decision-making may not only improve acceptance, but also partly compensate for the (perceived) inadequacy of current regulatory regimes. Accordingly, we aim to shed light on the ability and opportunities afforded to consumer-citizens to express their discontent with genome-edited food—as well as options available to proponents of genome editing and/or regulatory officials wishing to better take consumers' opinions and concerns into account. To achieve this, we apply and extend Hirschman's (1970) exit–voice framework to explore the contours of dissatisfaction and its related responses and then offer insights into a suitable governance approach for genome-edited food products. Hirschman's framework is unique in its potential to illuminate foundations of consumer and citizen engagement with products, producers as well as regulators. Applying the framework to the case of genome-edited food, we give specific attention to the use of labeling as a governance solution facilitating “exit” of consumers from markets, and to public deliberation as an expression of “voice.” As such, our analysis is grounded in the actual responses of consumer-citizens toward the potential market introduction of genome-edited food, whether this entails the controversy of GM food or more novel positions related to CRISPR-based systems. Instead of focusing on how acceptance of GM food can be improved (Araki and Ishii, 2015; Kolodinsky and Lusk, 2018), this framework thus institutes a “two-sided” understanding of governance. In specific, we contend that new regulatory approaches, whether in the form of labeling schemes enabling “exit” or deliberative mini-publics promoting a diverse, participatory type of “voice,” are crucial for ensuring that public viewpoints and concerns are taken into account in the political and social discussions of genome-edited food.

The article is structured as follows: in section Hirschman's Exit–Voice Framework and Its Application, we provide an overview of Hirschman's exit–voice framework and discuss some applications relevant to food consumption. In section GM Opposition and Genome Editing, we introduce genome editing in the context of opposition toward GM food. In section Current Debate on Governance of Genome-Edited Food, we briefly summarize the current state of the debate on governance of genome-edited food. In section Exit and Voice in the Context of Genome-Edited Food, we apply Hirschman's framework to the case of genome-edited food: after a general discussion of the role of exit and voice in this context, we analyse the manifestations of and preconditions for both. In section Conclusions, we offer conclusions.

## Hirschman's Exit–Voice Framework and its Application

### The Exit–Voice Framework

In his seminal book *Exit, Voice, and Loyalty: Responses to Decline in Firms, Organizations, and States* (Hirschman, 1970), the economist and social scientist Albert O. Hirschman enumerated a flexible framework for analyzing the diverse responses of consumers or users to dissatisfaction and discontent with the perceived quality of goods or services provided by private or public institutions. In this framework, a consumer/user has two main options to express dissatisfaction: exit or voice. Exit consists in refraining from consuming the good or service in question by, e.g., switching to a substitute good offered by another supplier. It represents, so to speak, a fundamentally market-based response as a result, and is thus broadly in line with conventional economic theory (Franzini, 2016; John, 2017). Voice, conversely, consists in various forms of expressing one's discontent in a way that directly reaches the producer/supplier, and specifically the management of the firm—whether through petitioning, protesting, lobbying, becoming more generally politically engaged, etc. It is thus a response that is more inherently political and participatory and which can be engaged in collectively or, more generally, “any attempt at all to change, rather than escape from, an objectionable state of affairs” (Hirschman, 1970, p. 30). In addition, this endows voice with a greater degree of flexibility and the ability to modulate how much dissatisfaction is expressed. Depending on the level of discontent, one can alternatively sign on to an petition; canvas directly to elected representatives; knock on doors in one's community to gather support; or sue the government or relevant firm if a problem is deemed sufficiently egregious. By going beyond just “voting with one's wallet,” voice thus provides one with the ability to convey more information than would be possible through exit alone.

With regard to the firms involved, or more generally those to whom dissatisfaction is addressed, Hirschman's framework also offers insights for how best to get their attention. In fact, one of the chief advantages of the framework lies in its ability to highlight mechanisms and opportunities available to individuals (as consumers and citizens) to push for changes in products or practices with which they are dissatisfied. First, it is crucial how the focus here lies on exploring the sub-par performance of firms, why this occurs, and how “temporary and remediable lapses” may be resolved. And, indeed, we highlight this phrase for how it signals these problems, as perceived by the individual consumers, to be (implicitly) understood as more or less correctable, assuming one uses the suitable mechanism or leverages sufficient pressure. Decisions about which strategy is most suitable for a given situation—i.e., gauging level of discontent, potential responsiveness of the institution, the number of viable substitutes available, how likely is a restoration of quality, etc.—are therefore crucial. Accordingly, at the center of the relationship between exit and voice we highlight a key trade-off: voice is more preferable when exit is not practical; exit more likely when transaction costs of voice are prohibitively high, or after prior efforts at voice have failed to bring about results. Moreover, though both represent responses to the declining quality of a good or service, the nature of the relationship between them—and thus which one is thus preferred—is likely to vary across contexts.^2^ Regarding public services, for instance, the availability of an exit option has actually been shown to foster further deterioration in service quality, even if this is the opposite of what customers intended. This may occur because e.g., the finances of bureaucracies are “insulated” from market pressure and not overly responsive to “market signals” or since those customers who opt to withdraw are often the ones with the necessary resources and expertise to ultimately enable change. Consequently, Hirschman (1970) concludes that state monopolies can, surprisingly, often be welfare-enhancing, e.g., if lack of exit options forces one to utilize another strategy to which bureaucracies are relatively more responsive: i.e., voice. Indeed, it is exactly in cases where customers are “locked in” that voice is most likely to be effective, namely as a way to compensate for the diminished reliability of exit. This example also makes clear how the level of competition in a given sector might represent a key determinant of the relative effectiveness of voice vis-a-vis exit: notably, if a customer only has a narrow set of alternatives for expressing her preferences, or the profitability of firms does not depend only on the quality of their offerings, then recourse to exit will likely be hamstrung.^3^ In this way, one can also observe the vital role of consumers/customers qua “agents of competition,” that is, assuming the necessary conditions are in place for them to perform it. On the other hand, exit and voice can and do complement each other in some circumstances: the threat of exit (instead of its silent realization) can lend powerful support to voice. For Hirschman, this makes exit “a last resort option that individuals do not want to take” (John, 2017, p. 515) in most settings, especially as this may prevent the departing members from reaping the benefits of any of those subsequent improvements in quality that their exit has made possible. Of course, this turns out to be less of an issue in market settings, e.g., the choice to switch from one product to another, where exit is most often “temporary” in nature.

### Exit and Voice in Subsequent Literature

In the ensuing decades, a large and diverse literature building upon Hirschman's framework has emerged. The framework has recently been applied to topics as diverse as behavior of farmers' associations in agricultural conflicts (Alpmann and Bitsch, 2015), the responses of communities of football fans to commercialization (Kiernan, 2017), vaccination policy (Geelen et al., 2016), maternal risk anxiety (Smyth, 2017), Euroscepticism in the European Parliament (Brack, 2012), and the persistence of Cuban socialism (Hoffmann, 2005). While Hirschman's own applications (e.g., Hirschman, 1978, 1993) and much of the literature have focused on the decline in “[political] organizations and states,” along with a sizeable body of research on exit and voice in the context of public services, relatively little attention has been given to exit and voice as strategies available to consumers (or consumer-citizens) in the marketplace. However, amidst the increasing contestation over food in the public discourse and a diminishing trust in food systems (Murdoch and Miele, 1999; Mazzocchi et al., 2008; Meyer et al., 2012), Hirschman's framework seems highly relevant. In the following, we briefly review a collection of the publications that have applied the exit–voice framework to the context of food consumption.

In spite of the relatively limited number of studies in this setting, the available literature turns out to not only be quite diverse but also to advance various improvements to the original framework. Light et al. (2003) deviate from Hirschman's original focus on *individual* responses to declines in quality and focus instead on “collective manifestations of voice.” They distinguish between two types of voice: *vertical*, directed at those responsible for the (perceived) deteriorations in product or service quality, and *horizontal*, directed at others who are “in the same boat” (Light et al., 2003, p. 477). They further note an example for each, namely organized protests and citizen/consumer associations, respectively. In this way, we grasp how the audiences for the two activities differ, with the latter seeking to build consensus among fellow consumers/citizens and the former speaking directly to power. This approach is then applied to early anti-GM protests in the US to underscore, *inter alia*, how voice was essentially the only option then available to consumers. According to the authors, this was explicitly tied to the absence of GM food labeling and, as a result, the absence of the necessary preconditions to render exit effective.

Meanwhile, focusing in particular on the rise in fair-trade certified products and vegetarianism, Newholm (2000) outlines the potential for and limitations of exit and voice as signaling devices available to ethically motivated consumers. Specifically underlining the insufficiency of exit as a “standalone” option for improving food systems, he notes that “peoples” [sic!] preferences cannot simply be read off their purchase behavior in the market' (p. 159). In fact, empirical studies have not been able to establish any direct link between motivations and attitudes, on the one hand, and consumer choices on the other (e.g., Bamberg and Möser, 2007; Grunert et al., 2014) and also that consumer behavior is not a good predictor of political attitudes and behavior (Hamilton et al., 2003). Newholm (2000, p. 161) instead argues that: “Consumer voice on the other hand, far from being unreliable, is the major source of business information,” while at the same time stressing that, at least in some cases, it is ethical concerns that are behind changes in buying patterns (that is, exit). In effect, the overall message is that, in the context of consumer ethics, not only are exit and voice both important but, in fact, given their varying strengths and weaknesses, they can be seen to be complementary to a large extent.

Representing a further step in this direction, Keeley and Graham (1991) have further argued that, actually, exit, and voice can each be disentangled into two distinct “values,” such that there end up being four possible constellations for responding to decline: *passive acceptance*; *internal change effort*; *quiet exit* and *vociferous exit*. In this way, they lay out not only how exit and voice might work together but also how this functions to diminish some of their respective shortcomings. For instance, they stress that the “trouble with exit, […] is that it permits firms to unfairly externalize system maintenance (feedback) costs by shifting these to exiting individuals—who may prefer (and, in fairness, deserve) a voice” (Keeley and Graham, 1991, p. 353). Informed by an empirical case analysis in the context of environmental risks, Zuindeau (2009) similarly proposes an extension of Hirschman's framework that disentangles active from passive responses as well as the implications of differing levels of dissent across groups (some dissatisfied, others satisfied). In Zuindeau's (2009) interpretation, exit can be counterproductive if the “exiteers” are those who had previously offered strong voice; conversely, voice can also be legitimizing and thus similarly undermining criticism. For this reason, he identifies four “key variables” able to influence the viability of exit and voice: the spatial extent of the problem in question; uncertainty around the problem; potential damages; and conflicts of interest involved. Rather interestingly, the last three variables are very similar to some points often stressed in conceptualizations of the precautionary principle, including in the context of GM food (e.g., Stirling, 2017). In fact, one can observe strong parallels between Zuindeau's characterization of “global risk,” i.e., its having unlimited spatial area, strong uncertainty, and very high potential damages, and the long-standing conception of DNA technologies in the literature on so-called technological hazards (Fischhoff et al., 1978; Slovic et al., 1985; Slovic, 1987). Particularly noteworthy in this context is Zuindeau's (2009) concept of informational voice, which takes the form of a request for information or a demand for further study and research that would lead to understanding the issue at stake better. In other words, voice ends up being modulated to the point that it is neither expressing a well-defined viewpoint nor seeking an outcome that is particularly clear-cut; rather, it wants to assert, if anything, that there still may be a degree of uncertainty around the underlying science, and perhaps that other “values” may also be required to come to an ultimate determination about its acceptability. In this way, we observe a type of voice that “defers” to the expertise of others, while nonetheless entreating them to take into account a broader perspective than might previously have been the case.

Before we apply those insights to genome-edited food and its governance, we first briefly review the history of GM food opposition and how genome editing differs from older GM techniques. It is crucial to underscore at this point, however, that the case of GM food serves as a probe or lens for exploring the broad category of genome-editing technologies applied in this domain. Instead of assigning undue importance to any one type of technology, we argue in the ensuing sections that there is a “systemic” component to much of the dissatisfaction that is expressed, and thereby rendering such criticism relevant though not necessarily specific to any given technology.

### GM Opposition and Genome Editing

There is a long history of public opposition toward genetically engineered food, particularly in Europe, where currently almost no GM food is being produced, or consumed (although it is fairly common to use GM feed in animal husbandry; Zilberman et al., 2018a). Before the advent of genome editing, skeptics focused mainly on environmental and health risks (Pirscher et al., 2018). There are multiple reasons for this. First, early GM techniques were rather imprecise, as it was not possible to determine exactly where a DNA snippet would be integrated into the DNA of the target organism. This thus gave rise to fear of unintended modifications and side-effects. Second, the focus was almost entirely on transgenesis, i.e., transmission of genes across the boundaries between species (or even kingdoms). Consumers have, however, been repeatedly found to be much more skeptical of transgenic than cisgenic food (Delwaide et al., 2015; Edenbrandt et al., 2018). Third, the vast majority of GM varieties was developed and sold by multinational companies such as Monsanto, DuPont, and Syngenta, which have long been viewed skeptically by consumers and civil society.^4^ Relatedly, GM food has been viewed, fairly or unfairly, as compatible mainly with highly intensive, industrialized, and environmentally harmful variants of agriculture (e.g., Gomiero, 2018). This is often linked to the fact that most commercial GM crops were bred for herbicide-resistance or *bt* (pest resistance) traits (Bennett et al., 2013) and that their use has led to pest resistances (Perry et al., 2016).

Genome editing, especially since the advent of CRISPR/Cas (Jinek et al., 2012), has significantly changed the picture across all three dimensions. First, genome editing is much more precise than earlier GM techniques, allowing for modifications of the genome at precisely specified locations and with few unintended, off-target mutations—though recently, a number of publications have questioned this claim (Schaefer et al., 2017; Haapaniemi et al., 2018; Kosicki et al., 2018). Second, the emphasis is more on non-transgenic modifications, including cisgenesis, targeted mutagenesis, gene silencing, and gene knockout (Bartkowski et al., 2018). Third, at least in the case of CRISPR/Cas, due to the low-cost of the technology's application and its higher flexibility, the heavy involvement of large multinational companies is no longer as essential as hitherto (Bartkowski et al., 2018). Accordingly, a shift in the public debate can be observed—today, environmental and health risks play less of a role; rather, the focus is shifting toward issues of naturalness, problem framing and, still, patents and property rights (van Hove and Gillund, 2017; Pirscher et al., 2018). Meanwhile, the general skepticism to GM food increasingly entails questions of the purpose of and need for “technical solutions” (van Hove and Gillund, 2017), a shift that also offers striking parallels to the older Golden Rice debate (Kettenburg et al., 2018). Drawing on the wide-ranging research of Sjöberg (2002), we could see all this as evidence of the increasing attention to the wider context in which technologies are introduced, implemented, and adopted. Instead of focusing only on perceptions of a technology like genome editing (and its associated hazards), this then draws into focus the relationship between the technology and its societal, scientific, and technological context in order to explore and understand attitudes toward risk. In fact, Sjöberg (2002) highlights three factors that are characteristic of the wider context of technology: whether a technology is readily replaceable, beliefs in the uncertainty of scientific knowledge, and the sense that its use represents “tampering with nature.” Not only is each shown to be among the most significant determinants for attitudes toward technologies, but they are especially impactful for gene technologies (Sjöberg, 2002). Moreover, the fact that all three are increasingly prominent in the shifting discussion of new genome-editing technologies also connotes that, as some of the more technical shortcomings of older-generation approaches are overcome, we can expect there to be more scope to consider the wider context to which technologies will have to relate—rather than their immediate and unequivocal acceptance. Thus, while the improvements offered by genome editing have the potential to change the public perception of GM food, the more short-term development is likely to be the greater engagement with novel types of arguments, and as a result, a continuation in the status quo where, at least in the EU, the majority of citizens remain skeptical of GM food (Twardowski and Małyska, 2015).

### Current Debate on Governance of Genome-Edited Food

The novel possibilities offered by genome editing, particularly CRISPR-based systems, have also brought to attention the shortcomings of existing regulatory regimes. For instance, as observed by Wolt et al. (2016), novel genome-editing techniques “do not readily fit current definitions of genetically engineered or genetically modified used within most regulatory regimes.” This has given rise to an (ongoing) debate about the proper governance regime for genome-edited food, as well as substantial differences in opinion, even among regulatory officials in various developed economies. For instance, in the US, the Department of Agriculture has decided that a gene-edited non-browning mushroom (*Agaricus bisporus*) can be cultivated and sold without any oversight, as it was created by “knocking out” the gene responsible for browning and without the introduction of foreign DNA (Waltz, 2016). More recently, the USDA has expounded upon this through its assessment that new genome-editing techniques, notably CRISPR, do not require any “special” regulation, specifically because these methods neither make use of nor rely on anything that may qualify as a “plant pest” (USDA, 2018). Conversely, albeit for somewhat distinct reasons, the European Court of Justice recently came to the conflicting determination that “all organisms obtained by mutagenesis” are identical in terms of their associated potential risks, and irrespective of any differences in the underlying technical process (ECJ, 2018). Not only does this raise the question of the appropriate level of regulatory scrutiny for the products of genome editing, as many commentators rushed to point out, but now there is the further issue of whether and how any “transatlantic” disparities in regulatory approach can be reconciled.

The broad debate on genome editing governance, which is likely to continue after the ECJ ruling, has been largely framed as the choice between product-based and process-based regulation (e.g., Hartung and Schiemann, 2014; Araki and Ishii, 2015; Huang et al., 2016; Sprink et al., 2016; Wolt et al., 2016). In the EU, what is currently employed best represents a process-based approach, implying that greater oversight is needed for any plant created using a technology classified as GM. However, arguing that non-transgenic genome editing “is by nature similar to the use of spontaneous variants or induced mutations in conventional breeding, with the advantage that only the desired change is introduced,” Huang et al. (2016) for instance “strongly advocate product-based rather than technology-based regulation” (p. 110). This would imply that most genome-edited crops would not be treated as GM products, and therefore should not be subject to the same regulations. Indeed, the advocate general of the European Court of Justice expressed a very similar viewpoint in his opinion to the court back in January (Purnhagen et al., 2018).

Such “evidence-based” or “science-based” approaches have been criticized as being founded upon the fallacious assumption that it is possible to make far-reaching societal decisions on an objective basis: “Empirical evidence matters, but human interpretation brings meaning to that evidence, and multiple perspectives can strengthen understanding” (Kuzma, 2016, p. 167). It has been further pointed out that “it is wishful thinking to believe that, by simply classifying products of NBTs [new breeding techniques] as non-GMOs, their commercial potential will be realized” (Malyska et al., 2016, p. 532). In fact, adoption and acceptance of novel products and technologies depends on both a range of stakeholders across the supply chain and a multitude of factors, some of which might not necessarily be deemed “objectively” relevant (Scheufele et al., 2007; Sarewitz, 2015; Baum, 2018). Malyska et al. (2016, p. 532) therefore contend that “the key issue is not whether new crop varieties are as safe as those developed by conventional plant breeding and thus fall outside the scope of current GMO legislation, but whether society perceives them as such.” In other words, the crucial issue is not whether there is definite evidence of a proof of an issue for human health or the environment, especially if there are widespread beliefs in the uncertainty of scientific knowledge (Sjöberg, 2002). Nor is the crucial issue even the pursuit of regulatory certainty, at least not for those actively engaged in developing and commercializing the new technologies. Instead, it is primarily a matter of public acceptance and legitimacy. Hence, what is most urgently required is a far-reaching societal dialogue on the (perceived) benefits and risks of genome editing, rather than one that only seeks to find technocratic “evidence-based” solutions (Jasanoff et al., 2015; Bartkowski et al., 2018) that draw upon and make use of only some types of evidence, perhaps to the detriment or ignorance of others.

### Exit and Voice in the Context of Genome-Edited Food

Adopting the perspective of a consumer-citizen, the distinction between exit, and voice as means to express discontent (and thereby offer feedback to producers/suppliers) only becomes relevant once genome-edited food products are already on the market. Before this time, there is nothing to exit from and, as such, any discontent about the *possibility* of market introduction can only be expressed by means of voice, as has been done for example in the debate spurred by the advent of CRISPR/Cas (section GM Opposition and Genome Editing).^5^ Given the various calls from those advocating for a product-based regulation that may facilitate the quicker introduction of (at least) non-transgenic genome-edited food products to the market (section Current Debate on Governance of Genome-Edited Food), the subsequent analysis thus orients itself around the counterfactual that genome-edited food is already available on the market. Whether this occurs because one country—e.g., the United States—has taken more immediate steps to “deregulate” such products or a few firms have shouldered the greater regulatory burden to bring the products to market is not so important—only that some products do exist on the market. For what follows, we will chiefly focus on two issues: the role(s) of exit and voice in the present context; and their manifestations and preconditions.

### The Role of Exit and Voice

The main role of both exit and voice is to express dissatisfaction and discontent with the existing state of affairs. As detailed in section GM Opposition and Genome Editing, many consumer-citizens have expressed their opposition toward GM foods in the past, by means of political protests and in choice-elicitation surveys.^6^ While genome editing, with its focus on non-transgenic modifications, may alleviate some of the public's concerns (Delwaide et al., 2015; Edenbrandt et al., 2018), there are still other concerns that go beyond health-related and environmental risks (van Hove and Gillund, 2017; Pirscher et al., 2018; see section GM Opposition and Genome Editing). Thus, it is fair to assume that, in the event of allowing genome-edited food products on the market, a significant and widespread level of concern and skepticism is likely to surface.

In this context, it is helpful to distinguish between two levels of dissatisfaction regarding GM food. First, there is product-related dissatisfaction, based particularly on perceived environmental and health risks. Here, we have to further distinguish between risks that are more private (health) or public in nature (environment), given that they have different consequences for the selection of response strategy. Notably, whereas exit is likely to represent a desirable strategy for risks that are perceived to be private, public risks cannot be sufficiently tackled in this fashion, because of the strong potential for externalities. Accordingly, I may be able to narrowly protect the health of myself and my family by means of exit (i.e., by not buying GM food), but if my main concerns center on the risks posed by GM crop cultivation for the environment, exit can neither solve the problem as long as production of GM food continues, nor indeed if the effects of this cultivation exert an indirect impact even on those not directly involved in their consumption. Here, voice is thus the potentially more effective strategy, as protection against these more public risks can only be achieved collectively—in the extreme, for instance, via a ban on activities like the cultivation of GM crops. In fact, it has been revealed that, once we enter in the context of public goods and externalities, market behavior only turns out to be loosely correlated with political behavior (Hamilton et al., 2003). Hence, the more voice-inflected forms of political activism prove to be a more appropriate strategy with respect to public-good concerns in the food context.

Second, there is the dissatisfaction more generally related to the food systems, of which GM and recently genome-edited crops end up being only one (perceived) manifestation of a broad, more symbolic bundle of (unwanted) characteristics (Gomiero, 2018). Bundled together, an observer of the current debate can thus find an assortment of issues such as market power, the shift toward industrialized, monoculture-based cultivation, distributions of property rights perceived to be unfair and, more generally, an unequal distribution of risks and benefits across groups within a society. This would suggest that, for at least some segments of the public, development and commercialization of GM food is understood to be entangled with the wider economic and societal circumstances into which these products would be introduced (see Sjöberg, 2002). Of course, it might be, and indeed frequently has been, objected that such perceptions are inherently biased, and thus in need of correction (cf. Stirling, 2008; Torgersen, 2009). Nonetheless, there are a variety of reasons to not simply dismiss such concerns out of hand, not least of which is the fact that the evaluations of experts have been revealed to severely underweight the importance to the public of socio-economic issues (Scheufele et al., 2007; Sarewitz, 2015). Attributing such concerns simply to “bias” would therefore run the risk of misunderstanding the reasons for dissatisfaction, not to mention the degree to which it exists.

More crucially for the role of exit and voice in the context of genome-edited food, it is necessary to recognize how broad societal, technological, and scientific conditions can incite not only an increase in the level of dissatisfaction but also prompt it to take one form over another. For instance, Schütz and Wiedemann (2008) have demonstrated how the risk perceptions of novel technologies are influenced by the identity of the beneficiaries. When a small- or medium-sized enterprise is most likely to benefit from their development, and not a multinational corporation, it is notable that people tend to assign lower risk probabilities to the likelihood for toxic damages, negative environmental impacts, and even those unknown risks yet to be considered. This speaks to the significance assigned to not just technology but rather the nature, scale, and identity of its introduction and implementation into (existing) economic systems. Similarly, Betten et al. (2018) find, somewhat contrary to expectations, that most people are neither inherently for nor against synthetic biology; instead much of the criticism stems from core values about the relationship of society with science and technology as well as general feelings of discontent with the prevailing context. As such, if there is anxiety about wider trends in technology development, for instance because of the potential impacts for employment or the greater prospect of firm consolidation, such anxiety might then manifest itself as an ostensibly “irrational concern” about one specific technology, that is, because it is not only not viewed as a potential solution but rather as something that could make things worse. Broadly speaking, the crucial point is that *it is not necessarily the technology itself that arouses societal unease but rather its (perceived) engagement with existing socio-techno-economic systems* (Jasanoff et al., 2015; Baum, 2018).

With regard to expressions of voice and exit, moreover, this also opens up the specific possibility that what I may wish to exit from, or raise my voice against, is not a particular product but rather the whole food (production) system. Thus, while it is clear that exit and voice are supposed to communicate discontent and dissatisfaction, in the domain of food, the reasons for discontent and dissatisfaction are potentially greater and more complex than in many other areas. Of course, this need not be unique to the food sector and yet, whereas genetic engineering has been deemed acceptable if used for other purposes, notably pharmaceuticals, and plant protection (Frewer et al., 1995; Knight, 2006; Christoph et al., 2008), applications to food have frequently “amplified” the controversy of novel genome-editing technologies (Frewer et al., 2002; Pidgeon et al., 2003). As a result, there are many reasons to believe that the evolution of discussions in the food sector could follow their own unique logic. On the one hand, we see burgeoning interest in many developed countries regarding the quality and provenance of the food one eats and growing appreciation of environmental, health-related, and socio-economic impacts of conventional food systems. Given that this is coupled to the advent of innovative arrangements such as organic food, farmers' markets, community supported agriculture and fair trade, there is a change in both the quantity and quality of consumer involvement within the food sector. Reflecting the increasingly diverse, multi-dimensional responses available, for instance, a person who is “fed up” with established food systems can express their dissatisfaction by buying less from a given firm; protesting the particular activities with which they take issue; supporting related policies by contacting their representative; or, at a more systemic level, “voting with their wallets” by frequenting farmers' markets or becoming a member of a box scheme, rather just switching brands or choosing to buy organics. Which of these available strategies would best be able to not only express dissatisfaction but also inspire a desirable reaction by those in charge, however, depends on whether a variety of preconditions are in place to ensure their effectiveness.

### Manifestations of and Preconditions for Exit and Voice

As already indicated in the previous sub-section, exit and voice can communicate discontent and dissatisfaction in a variety of fashions when it comes to food in general and genome-edited food in specific. Given the range of manifestations that may emerge as a result, it is crucial to explore the extent to which, depending on the specific context and purpose of the activity, the conditions and requirements of success could vary. For instance, if the existence of alternatives is necessary to render exit effective, the increasing manifestation of such activities is unlikely to take place in the absence of broader changes. Rather, we would expect reliance on exit to occur in response to the availability and diversity of alternatives on offer. And, if the scope of dissatisfaction is linked with established food systems at large, then the alternatives would have to be of a similar type as well—that is, alternative food systems.

#### Exit

As suggested above, dissatisfaction in the food sector can occur at two distinct levels: the level of products and the level of systems. In this respect, we see one of the crucial ways in which this sector represents a clear departure from others that have previously engaged the attention of exit–voice researchers (section Hirschman's Exit–Voice Framework and Its Application). Indeed, exit has typically been understood as a strategy that is more relevant at the level of products, for instance, because of the way that we individually bear any related risks (and collect the benefits) of our food choices. Further undermining our capacity to express dissatisfaction with entire food systems, there is the added impracticality of “exiting” the food system of a country, by opting for instance to purchase all food from Canada instead of the United States if the former were to adopt GM labels or support family farmers. Aside from leaving the country, this would leave critical consumer-citizens with “nowhere else to go.”

However, as alternative food systems have become available, the scope of choice that is afforded to consumer-citizens even at the level of systems has increasingly grown. Organic agriculture is often perceived as one such system (Reganold and Wachter, 2016; Gomiero, 2018), specifically as it positions itself as a solution to the perceived deficiencies of the industrialized, environmentally harmful, and excessive concentration of conventional food systems. Of course, it should be noted that the degree to which it is in fact a clear alternative, at least in terms of environmental impact, has been called into question (e.g., Meemken and Qaim, 2018; Tal, 2018). Even if the large scale forms of organic agriculture may not drastically differ from existing approaches, there are others adopting a more regional character, e.g., through community-supported agriculture or other “independent” arrangements, so alternatives do exist, thus offering a greater degree of “exit potential” for consumers who wish to extricate themselves.

In any case, exit generally requires the capacity to distinguish between alternatives. In the more extreme case of “complete” exit from conventional systems, buying only (regional) organic food could signify a viable option as “[o]rganic management systems do not use genetically modified organisms (GMO) or their derivatives, except vaccines, in all stages of organic production and processing” (IFOAM, 2017). However, taking such recourse would not only require the complete detachment from conventional food systems but is also likely to be quite costly, both financially (Seufert and Ramankutty, 2017) and in terms of the effort needed to identify and purchase food of a suitable quality. Moreover, given the increasing specialization and “industrialization” of organic farming, the potential of this strategy to serve as a way to escape the conventional food system is somewhat diminished. As it becomes more and more difficult to differentiate “authentic” organic producers, i.e., those who inhabit the original ideals of the system, from those who do the bare minimum to attain the desired premiums, ever more effort and attention is required to make an informed decision. This represents, in fact, a long-standing issue in the literature on consumer welfare, notably, the requirements for choice that is actually “free.” On this point, and proposing his deeper understanding of what constitutes consumer sovereignty, Scitovsky (1962) has drawn a strong distinction between the ability of markets to cater to so-called “minority preferences” and “majority preferences.” Pointing to the often-unattended downsides of pursuit of economies of scale, he observes that what tends to pass for “variety” in satisfying the desires of most people turns out to signify an illusory choice offered among products that are distinct only superficially, and mostly identical in their core characteristics. As a result, this offers any consumer with somewhat atypical tastes not only an increasingly narrow set of alternatives on which to express their preferences but also more limited prospects to pursue genuine “exit” if this is deemed to be desirable. Limitations on the range of alternatives that are genuinely distinct, that is, not just in relation to a few peripheral features but also for domains of more fundamental importance to society, environment, etc., are thus a radical constraint on the effectiveness of exit.

While Scitovsky and others (e.g., Sirgy and Su, 2000) are able to sketch the wide context in which consumers become less sovereign, this literature focuses less on how this impacts the actions and decisions of the consumers themselves. For this, we must look at Hirschman (1970), specifically in relation to the archetype of “quality connoisseurs.” In specific, these individuals are both most accustomed to a high level of quality and, accordingly, more likely to be disappointed with declines in product quality. One potential explanation for this would pertain to the growing complexity and uncertainty involved with ascertaining the quality of products within modern economies, not least because of the growing technological sophistication even in the area of food production. As such, if one had a background in microbiology, they might then be (given suitable levels of time and interest) more able to research the competing claims about the safety and efficacy of genome-editing technologies than someone who is less expert. In fact, according to Hirschman (1970), such actors play an essential role for the broad operation of exit and voice, e.g., from their greater willingness to engage in “opinion leadership” by assembling their fellow citizens or directly reaching out to management. At the same time, if there is a higher-quality but more expensive substitute, these people are just as likely to abandon the firm and exit in favor of this alternative.^7^ We therefore observe that such individuals are more likely to engage in exit and voice: the desire for this quality occurring even and in spite of the costs of doing so.

However, even if these “connoisseurs” are more likely to be motivated and willing to retain their desired level of quality, this alone is no guarantee that they will actually be able to do so. On the one hand, this is a result of the tendency of there to be a relative paucity of alternatives at the higher-quality end of markets. Contrasting with the more typical clustering of products at the low-quality, low-price end of the spectrum, it turns out that the choosiest may necessarily have less to choose from. Even if they have a greater opportunity to leave, this lack of choice can thus serve as a check on the speed with which connoisseurs will exit in favor of the greener pastures elsewhere. In other words, possessing a greater amount of resources offers no guarantee that this is matched by an increasing quantity of alternatives, nor even a larger product assortment than those with fewer “opportunities.” As a general point, likelihood of engaging in exit thus reflects the trade-off between the number of suitable alternatives that exist and the quality preferences of individual consumers.

How then are consumers who might favor “exit” likely to respond? On the one hand, it may be argued that, even if complete exit is not feasible, a more “partial” exit, that is, one that balances personal costs against social benefits, can still have an impact. For instance, dissatisfaction with the quality of the offerings of a firm (or the entire system) could lead a consumer to reduce their amount of consumption, e.g., by purchasing from another firm or, in the case of the entire food system, frequenting more farmers' markets or even starting a home garden. In the latter case, we therefore find one of the few “true” alternatives for exiting from the current food system, notably, substituting one's own production and/or just consuming less overall. Nonetheless, to the degree that the concerns of an individual are public in nature, it turns out that any kind of “individual” exit only represents an imperfect solution, for reasons similar to those discussed above. That is, whenever the impacts of food production affect the quality of “public goods,” of which the environment is perhaps the clearest example, these are necessarily diffuse and non-exclusive in nature. For this reason, even if one is able to “escape” having conventionally produced food on one's table, it is not possible to escape the negative externalities of conventional food production in a more general sense. In the words of Hirschman (1970, p. 104), this results in a situation where “[i]n spite of exit one remains a consumer of the output or at least of its external effects from which there is no escape.”

Instead of cause for cynicism, this leads Hirschman to explore alternative ways in which exit can effect change, notably, by ensuring that one's exit directly contributes to desired improvements. Recognizing that there in fact limitations on individual exit, greater emphasis is therefore placed on the *timing* of one's exit, i.e., to ensure that it not only expresses dissatisfaction but is effective in doing so. For someone to best avoid hypothetical damages, it could then turn out to be useful to forestall exit as long as possible, thereby guaranteeing one retains a modicum of influence to be exercised from within. In the words of Hirschman (1970), this however results in a shift in the reading of the situation to where “the alternative is now not so much between voice and exit as between voice from within and voice from without (after exit)” (p. 105). In such a scenario, we might conceive of “exit” within the prevailing system as basically recurring over time, by taking the form, e.g., of a consistent choice of the brand that attempts to minimize its harmful impacts or of the exact offering of a given brand that best satisfies consumer concerns.

Besides signaling the rather limited scope for “exit” in this context, the foregoing highlights how the effectiveness of exit depends, first and foremost, upon the ability to distinguish between, e.g., genome-edited and non-genome-edited products. Having labels on GM food is thus one of the preconditions for choice to be effective, including for products that are “only” genome-edited. Contrary to calls for the product-based regulation of genome-edited, non-transgenic crops, we thus note that there are also more informational and expressive reasons for adopting a process-based approach. In other words, if consumers perceive such labels as useful for making choices, regardless of whether they see the underlying technologies as problematic, their mere absence could raise “red flags” where none were present before. At first glance, this might seem counterintuitive; however, there is growing evidence that, not only are attitudes toward GM food not affected by the existence of labels (Kolodinsky and Lusk, 2018), but that their absence could spark concern, even for those who might be more likely to accept such products. Instead of labels possibly prejudicing the public against genome-editing technologies, it could be their absence which proves to be more of an issue if individuals are indeed to be asked to make informed decisions.

While useful, it still remains that labels ought not be taken to be “sufficient” for the effectiveness of exit, especially given the range of other factors involved. Some of these have been addressed in the rather extensive literature on the economics of labeling (McCluskey et al., 2018; Zilberman et al., 2018b). A particularly important question is “What should be labeled?” There has been a proliferation of labeling schemes in recent years, all of which, by claiming to provide different kinds of information, both relevant and irrelevant, induce a constant risk of information overload (Verbeke, 2005). In this way, consumers are confronted with the “paradox of choice,” whereby the overwhelming amount of alternatives and products, while generally assumed to be beneficial by standard economic theory, ends up reducing welfare (Schwartz, 2004). According to Scitovsky (1962), for choice to actually be “sovereign” and free, it is necessary for consumers to actually be able to evaluate the alternatives available—also without this requiring them to invest an unreasonable amount of time or energy to do so. Accordingly, if labels fail to clearly distinguish products in ways that the public can understand, e.g., by allowing too many exemptions or creating multiple levels of “non-GMO,” or make it difficult for certain groups to track down relevant information, e.g., by solely employing QR (or quick response) codes, this would undercut their ability to support decision-making. Partly owing to such shortcomings, the quality perceptions of labels have been shown to vary across contexts. Indeed, perceptions of healthiness and sustainability have been tied to the type of retail format where products are sold (van Rompay et al., 2016; Baum and Weigelt, 2019). If, however, the value of an organic label in a supermarket exceeds the value of one in a discounter format, labels are then no longer able to convey the same information in all situations, or to serve as an unbiased basis for information provision. Indeed, it has been illustrated that many consumers therefore question the reliability of organic and fair-trade labels (Jahn et al., 2005; Janssen and Hamm, 2012), even going so far as to dismiss them as “marketing tools” that fail to provide what is promised (Rousseau, 2015).

From the perspective of the exit–voice framework applied here, there is one general problem with exit that cannot, however, be solved by labeling, no matter the accommodations that are made: if the aim is to signal dissatisfaction with specific characteristics of a product or production system, exit turns out to be of very limited relevance since it is an imprecise signaling device. Producers usually do not know why exactly it is that a consumer decides to exit, given that exit is carried out in relation to the product (or system) in full (Hirschman, 1970; Newholm, 2000). In addition, use of exit as a standalone strategy suffers from the same fatal flaw of collective-action problems: that is, change in and of the system (here: food system) cannot be triggered by the actions of one individual. Not only is there the potential for firms/institutions to ignore the activities of any one individual (or handful of individuals), there is the further problem that such activities, instead of giving rise to a “virtuous circle” where other consumers opt to take part, might just as well trigger a higher incidence of free-riding behavior. As exit is ex definitione an individual-level strategy, it thus requires the complement of other strategies to be a contribution to collective action. Enter voice.

#### Voice

Having outlined the manifold limitations to the effectiveness of exit, the foregoing might provide the impression that consumers are increasingly “captive” to commercial interests. Almost 20 years ago, Sirgy and Su (2000) thus asserted that the capacity of “sovereign” consumers to exercise an unconstrained freedom of choice has now become “more of a fiction than a fact.” In specific, the authors note, *inter alia*, the diminishing expertise, motivation, and opportunity of individuals to make decisions broadly in the interests of societal welfare to explain why they are unable to hold firms to account. Given the strictures of “an increasingly high tech world,” they then propose that consumers are replaced by the wider set of stakeholders as ultimate arbiters of business performance—thereby absolving the former of any specific, deeper responsibility. Consumers are thus no longer treated as sovereign, but simply another actor group whose interests must be considered when making decisions of broadly societal relevance.

Holding to our stated aim of facilitating a more active role for the public, we however call into question the validity of their conclusions. Firstly, the tendency to neglect the average citizen and her interests, or to suppose that engaging in “self-regulation” on their behalf is sufficient, is often one of the broad complaints lobbed against the established system of food production, and as a result against the commercialization of genome-edited products (Stirling, 2008; Torgersen, 2009; e.g., Jasanoff et al., 2015). Furthermore, the foregoing seems to suppose that, should individuals be limited in their efficacy as consumers, they would then have no other recourse for making their dissatisfaction known. Conversely, the dialectic of the exit–voice framework elucidates that, if use of exit is forestalled, this opens up a greater likelihood to focus attention on opportunities to engage in voice (Hirschman, 1970, pp. 70–72). Throughout his examination, Hirschman (1970) focused mainly on those voice options available to individuals that are not institutionalized: be it protests, boycotts, petitions, letter-writing campaigns, etc. In the context of genome-edited food, we can therefore see manifestations of voice through, for instance, the widespread “March(es) Against Monsanto”—which first emerged, in fact, in response to the failure of a ballot initiative in California that would have required GM labels on food products—and omnipresent petitions, whether from consumers, scientists, and non-governmental organizations (NGOs), urging firms and regulatory agencies to label or outright ban these products. Of course, given the strong differences in opinion here, it is unsurprising that “counter-petitions” pronouncing the safety and desirability of these products are also widespread—best exemplified by the widely-publicized letter “supporting precision agriculture (GMOs),” signed by 135 Nobel laureates, and identifying efforts of Greenpeace against GM crops as a potential “crime against humanity.”

While the fraught nature of the debate should come with little surprise, these examples are useful for a few reasons. First, they illustrate both the prevalence and variety of manifestations of voice, not to mention the diverse actors who could engage in such activities. For instance, in addition to the “voices” of consumers, there are many recent cases of leading experts also speaking out for, e.g., a moratorium on human germline editing by means of CRISPR-Cas (Baltimore et al., 2015) and a ban on the field-testing and development of gene drives until “open and international discussions” have an opportunity to occur (Esvelt and Gemmell, 2017; Noble et al., 2017). In fact, owing to the way that public knowledge about new technologies tends to substantially lag behind that of experts, the initial expressions of voice are most likely to emanate from those with the most experience working with and developing them. Second, the examples also point to a crucial limitation on the exercise of voice, which is of importance given that it extends to Hirschman's framework more generally. Notably, all the various manifestations mentioned here, while clearly able to express the dissatisfaction of the consuming public, fall short of supporting a more direct engagement with the relevant decision-making processes. From the perspective of the *governance* of genome-edited food, the more relevant issue is thus how the voice option can be institutionalized in order to be more accessible for critical individuals and groups wishing to have recourse to it. Institutionalization of voice in this manner is crucial, in that it secures the deeper embeddedness and integration of voice within political decision-making processes and mechanisms, thus allowing expressions of voice to be more effective (as its addressees are likely not only, or not even mainly, the producers of genome-edited food but rather regulators). On the one hand, we observed in section The Role of Exit and Voice that many of the concerns surrounding GM food (including genome-edited food) are public in nature; therefore, their (re)solution requires collective action. What is more, given the (perceived) deficit of legitimacy in this domain, it is principally crucial to engage with and integrate the public more deeply—especially with the limited effectiveness of exit here (section Exit). With these facts in mind, we would like to step beyond Hirschman's original framework and introduce some insights from the theory of deliberative democracy that may be instructive in the present context.

Starting off broadly, Jasanoff et al. (2015) have already emphasized the importance of public deliberation among stakeholders for reaching a legitimate solution to the controversies enfolding genome-edited food. Especially given the extent of the stakes involved, going beyond particular breeding techniques to also encompass more fundamental questions regarding the future of the food system, a broad debate on applications of genome editing to the food domain, embedded in a more general debate about the food system as a whole, appears warranted. As already indicated above, the parties to the current GM food debate currently confront each other in a quite antagonistic fashion, which may be interpreted as an instance of “deep moral disagreement,” i.e. a situation in which “parties to a dispute do not recognize the legitimacy of each others” [sic!] values' (Dryzek, 2013, p. 337). In such a case, public deliberation can serve as a useful tool to uncover a “normative meta-consensus” that would allow the parties to at least recognize the legitimacy of each other's positions (without necessarily agreeing on a specific course of action) (Dryzek, 2013). The existence of this kind of mutual respect on both sides represents a fundamental precondition for the more effective use of voice in this context, even if this ideal is rarely fulfilled in reality. When looking for instance at the nature of the discourse from Greenpeace and other non-governmental, civil-society organizations on the one hand and notable advocates of GM food on the other, it is readily clear that any kind of meta-consensus is presently lacking. We have already mentioned the charge levied against the activities of Greenpeace by the assembled Nobel signatories above; in addition, note the title of an influential paper from the “father” of the Green Revolution, Norman Borlaug (2000): “Ending World Hunger: The Promise of Biotechnology and the Threat of Antiscience Zealotry.” On the other side of the divide, activists tend to attack scientists voicing pro-GM opinions as being allegedly paid by Monsanto.

This shows that while the importance of civil society as a herald of collective voice cannot be overemphasized (Habermas, 1996), in situations of deep moral disagreement additional institutionalized voice options and a deliberate broadening of the debate are needed. Examples of successful deliberative processes in deeply divided societies such as Northern Ireland (Luskin et al., 2014) demonstrate the broader potential for institutionalized deliberation to help bridge even strong differences of opinion. In the context of genome-edited food, Bartkowski (2019) discusses using deliberative mini-publics, i.e., moderated small-group discussions including testimonies by expert witnesses, to facilitate a societal process aiming at shared understanding. It has been argued that such mini-publics can be a helpful complement to conventional, representative-democratic political processes by contrasting “majority opinions” (e.g., the widespread skepticism toward GM food, including genome-edited products) with such shared understandings reached by small, in-depth group discussions of representative samples of the population (Lafont, 2017). In fact, experimental results suggest that mini-publics can influence public opinion (Ingham and Levin, 2018). However, careful design is necessary for such deliberative institutions to work properly (Aasen and Vatn, 2013): for instance, the case of UK's 2003 “GM Nation?” public debate has been invoked as a negative example because participation was based on self-selection (Goodin and Dryzek, 2006), thus potentially leading to biased results.

As showed by the recent decision of the ECJ, there is urgent need for new GM legislation that is up to the task of dealing with genome editing. At the same time, there is a need for a rational debate among the parties, including not only biotechnology companies, scientists and anti-GM NGOs, but also the broader public. Innovations such as mini-publics might help institutionalize voice and thus offer consumer-citizens an opportunity to participate more actively in the debates currently characterized by deep moral disagreement. Moreover, such institutionalized voice opportunities have the potential to generate more understanding of the underlying motives of the participants, including general dissatisfaction with the modern food system. Last but not least, if properly institutionalized, mini-publics may help legislative bodies navigate the complex, morally charged field of GM regulation so as to identify legitimate solutions to the currently inadequate (in face of genome editing) GM law. In fact, public consultations have been applied to inform the EU agricultural policy, and stakeholder consultations are already part of EU's GM food chain governance (Bengtsson and Klintman, 2010).^8^ Strengthening and extending these institutions by the inclusion of more deliberative elements, possibly also in cooperation with the European Parliament as the democratically legitimized legislative body of the EU, would be a viable step toward resolving the controversies of genome editing.

## Conclusions

In this paper, we have applied and extended Albert Hirschman's exit–voice framework in order to shed light the proper governance of genome-edited food. Starting from the premise that it is not the type of governance approach that matters most but whether governance proves suitable to not only enable consumer-citizens to express and react to sources of dissatisfaction but also open up space for the public to assume a more active role, we analyzed the dominant expressions of voice and exit in relation to genome-edited food. We specifically argue, first, that opposition in many cases signals the existence of a deeper dissatisfaction with conventional food (production) systems and their negative externalities: for environment, society, human health, and animal welfare. Criticisms about GM food, for instance, are not therefore specific to any one technology or product application, but rather share aspects that are consistent across all others that highlight and draw out similar concerns. Second, we posit that much dissatisfaction with and skepticism toward the biotech industry could thus reflect the lack of effective recuperation mechanisms, whether exit or voice. As a result, what is perceived as unfair or misplaced criticism—from the point of view of proponents and actors in the food industry—could represent a delayed response on the part of consumer-citizens to previous grievances, specifically because of their previously limited outlets available to them for expressing their dissatisfaction. Also, calls from both science and industry to reduce options of exit (via product-based regulation) might well contribute to the dissatisfaction. If this is the case, improvements in the availability of exit and voice could go a long way to also reducing the levels of “unfair” criticism. Based on these points, we considered possible manifestations of exit in this context as well as the conditions that are required for these strategies to be effective. Ultimately, we conclude that, in situations where dissatisfaction extends to the food system as a whole (channeled as a result into the opposition toward GM food, among other things), exit turns out to be of limited relevance. In part, this is a reflection of the nature of the problems themselves, most notably, that the “goods” (or “bads”) in question do not just affect discrete individuals but are instead more public in nature. As a result, the ability to find solutions not only eludes the grasp of a single individual, instead requiring that collective action take place, but it will also be difficult for any individual to completely “isolate” themselves from the wider consequences of the system in place. In other words, consumers can select the types of food they serve for dinner but not whether or not the environmental or societal consequences of the food system (if any) have an impact on their daily lives.

Nonetheless, although exit is only an imperfect strategy, it is still likely to be relevant in some contexts, most notably for influencing the decisions of certain firms. For exit to serve as a viable option in this regard, it must be possible for consumers to distinguish between the alternatives on offer—thus making labeling a necessary condition. This does not mean, however, that labeling is *per se* sufficient for effectively expressing dissatisfaction across all contexts, not least because of the risks of information overload and often-circumscribed variety of alternatives from which individuals are able to choose. Given the limitations on the exit option, we therefore turned to voice and, in line with our aim of studying options to foster more institutionalized forms of action, we extended Hirschman's original framework by introducing some insights from the theory and practices of deliberative democracy. We emphasized the deep moral disagreement that characterizes the current state of the debate on GM food (including genome-edited food) and stressed the potential of institutionalized voice (e.g., deliberative mini-publics) to diversely inform and orient a more wide-ranging societal debate into genome-edited food and, more broadly, the future of the food system. We see potential to extend existing institutional structures in the EU to enable institutionalized voice and contribute to crafting new GM food regulations, adequate for genome editing technologies.

The foregoing conceptual analysis, however, leaves many questions open, partly given its reliance on a few requisite simplifications. For instance, we have ignored the distinct variants of labeling approaches (mandatory vs. voluntary, governmental vs. self-declared vs. third-party), as these are both less important for the present analysis and, moreover, covered in much greater detail in the relevant literature (e.g., Zilberman et al., 2018b). Nevertheless, further analysis of the comparative strengths and weaknesses of the varied approaches against the background of our findings would be interesting and informative. With regard to voice, we have implicitly assumed a rather idealized account of deliberative democratic institutions. There is, in fact, a large literature that highlights the limitations and weaknesses of such practices, such as the constraints of power dynamics and the unclear role of emotions (Mendelberg, 2002; Chilvers, 2009). Nonetheless, what specific consequences these limitations have in the context of genome-edited food must be left for future research. Perhaps most fundamentally, there is a deeper need for information about the types of conclusions that institutionalized voice—whether by mini-publics or some other format—can reach in the context of genome-edited food, as well as how these may best be used to inform and orient public policy. Further research in this vein is urgently needed.

Last but not least, assuming that a product-based regulatory approach is not ultimately deemed to be democratically legitimate, there are many questions about which kind of governance regime could best balance the benefits and costs of genome-editing products in the food domain. Indeed, the recent judgment by the ECJ (2018), by lending support toward further risk assessments and value-based discussions, is much more likely to represent the beginning of a wider debate into this topic than offering the last word. In this regard, we contend that further progress in application of the exit–voice framework here can prove useful by, *inter alia*, helping to establish the preconditions and institutional forms necessary for such strategies to be able to effectively express (and resolve) the sources of popular dissatisfaction with the food sector.

## Author Contributions

All authors listed have made a substantial, direct and intellectual contribution to the work, and approved it for publication.

### Conflict of Interest Statement

The authors declare that the research was conducted in the absence of any commercial or financial relationships that could be construed as a potential conflict of interest.

## Footnotes

^1^i.e., the targeted modification of single base pairs in the DNA. In EU law, conventional, non-targeted mutagenesis (via exposure to radiation or acids) is however exempted from GM regulation, notably, owing to the fact that it has been “traditionally used without proven harm for public health or the environment” (ECJ, 2018, p. 3).

^2^Conversely, Hirschman underscores that the dynamics of what he calls the “management reaction function” could conspire to put the firm beyond saving, no matter the level of pressure exerted. Notably, a mismatch between the timing of consumer action and ability of the firm to respond, perhaps due to competition in the relevant sector, could result in the needed feedback coming too late. For instance, if customers are slow to respond to a change in quality, before then doing so *en masse*, by the time management receives this information, it might be too late to engage in the necessary remediation that would stem the tide of customers leaving.

^3^Indeed, the relative effectiveness of a given option also depends on the type of decision involved, i.e., not only the availability of substitutes but also potential costs of making a wrong choice. On this point, Hirschman (1970, p. 41–42) underscores that “the sheer number of available goods and varieties in an advanced economy favors exit over voice, but the increasing importance in such an economy of standardized durable consumer goods requiring large outlays works in the opposite direction.”

^4^A significant exception is Golden Rice, which was developed in a non-commercial context (though Syngenta has been involved), but has been still targeted particularly by environmental NGOs such as Greenpeace. For an in-depth study of the Golden Rice debate, see Kettenburg et al. (2018). For a critical discussion of why Golden Rice has not been widely adopted, see Stone and Glover (2017).

^5^In this regard, the facility and relevance of comparisons/contrasts to other technologies, with genetic engineering serving as perhaps the most notably, represent a crucial basis for being able to produce the type of counterfactual “analysis” that will allow decisions and criticisms in line with the values which one espouses.

^6^In absence of marketed GM food in the EU and of GM labeling in the US (except for Vermont), no opposition could be expressed via market behavior.

^7^Here it is useful to note that the reasons for doing so are not explainable in terms of the (looser) budget constraint alone. Rather, according to the exit–voice framework, this is a matter of retaining the level of quality to which they are accustomed and, what is more, the difficulty of achieving the same outcome by means of voice alone.

^8^However, Bengtsson and Klintman (2010) note that a major problem of the stakeholder consultations by the European Food Safety Agency (EFSA) and the Directorate General for Health and Food Safety (DG SANTE) is that they do not include the general public in their concept of “stakeholders.”
